# Supplementary material for: How protein hydration depends on amino acid composition, peptide conformation, and force fields
Source: Biophys J. 2025 Nov 27;125(1):255–69. doi: 10.1016/j.bpj.2025.11.2683 (PMC12821034; doi:10.1016/j.bpj.2025.11.2683)
Supplement: Document S1. Figures S1–S14 [file mmc1.pdf]

Biophysical Journal, Volume *125*

Supplemental information

How protein hydration depends on amino acid composition, peptide conformation, and force fields

Johanna-Barbara Linse, Tobias M. Fischbach, and Jochen S. Hub

Supporting Information for: How protein hydration depends on amino acid composition, peptide conformation, and force fields

Johanna-Barbara Linse, Tobias M. Fischbach, and Jochen S. Hub*

Theoretical Physics and Center for Biophysics, Saarland University, 66123 Saarbrücken, Germany

E-mail: jochen.hub@uni-saarland.de

Supporting Information Methods

Maximum-entropy refinement with the XAO peptide ensemble against SAXS data

To obtain a set of 20 XAO conformations that are representative for the XAO solution ensemble, we carried out SAXS-restrained ensemble simulations with commitment to the maximum entropy principle.[1] Four parallel XAO simulation replicas were coupled on-the-fly to the SAXS curve taken from Ref. 2. Simulations we carried out with GROMACS-SWAXS, version 2021.5, as freely available at https://gitlab.com/cbjh/gromacs-swaxs. Documentation for GROMACS-SWAXS is available at https://cbjh.gitlab.io/gromacs-swaxs-docs.

Four starting structures for the SAXS-restrained simulations were taken from the XAO ensemble refined against NMR data by Makowska et al.[3] and set up for simulations as

described in the Methods. To couple the simulations to the SAXS data, SAXS curves were computed from the simulations on-the-fly using explicit-solvent SAXS calculations, thereby taking scattering contributions from the hydration shell into account.[4–6] SAXS curves were averaged on-the-fly using a memory kernel that decays exponentially into to the past using a memory time of 100 ps [molecular dynamics parameter (mdp) option `waxs-tau`]. A q range from 0.065 Å^{-1} to 0.58 Å^{-1} with 30 equally-spaced q-point was used (mdp options `waxs-startq, waxs-endq, waxs-nq`). The SAXS curve was updated every 125 ps (mdp options `waxs-nstcalc` together with `dt`). A force constant of unity was applied, and the restraints were turned on gradually over 10 ns (mdp options `waxs-fc, waxs-t-target`). During the simulations, and prior to computing SAXS-derived forces, the experimental SAXS curve was fitted to the calculated curve via $I_{exp,fit}(q) = f \cdot I_{exp} + c$, by minimizing χ^2 with respect to the calculated curve. Here the factor f accounts for the overall scale, and the offset c accounts for a putative uncertainty from the buffer subtraction. No fitting parameters owing to the hydration layer or excluded solvent were used, implying that also the radius of gyration was not adjusted by the fitting parameters. The agreement of the SAXS curve obtained from the refined XAO ensemble with the experimental data is shown in Fig. S1.

SAXS-restrained simulations were carried out for 150 ns. The spatial envelope was built at a distance of 12 Å from all XAO atoms during free simulation that started from four different structures. Solvent atoms within the envelope contributed to the calculated SAXS curve as described previously.[5] The temperature was controlled at 298.15 K using a stochastic dynamics integrator.[7] All other simulation parameters were chosen as described in the Methods.

From the four trajectories collected from the four parallel replicas, five configurations each were taken from the simulation times 30 ns, 60 ns, 90 ns, 120 ns and 150 ns, thereby providing 20 independent conformations. These conformations were mutated as described in the main text and used for follow-up SAXS calculations. During follow-up simulations, these 20 conformations were maintained by applying positions restraints either to the backbone or

to heavy atoms, as described above.

On the calculation of the radius of gyration R_g^{Prot} of the bare protein

In this study, the radius of gyration R_g^{Prot} was computed from the coordinates of the atoms $\mathbf{r}_i$, weighted by the number of electrons of the atoms n_i:

$$(R_g^{Prot})^2 = N^{-1} \sum_i n_i (\mathbf{r}_i - \mathbf{r}_{com})^2,$$

where $\mathbf{r}_{com} = N^{-1} \sum_i n_i \mathbf{r}_i$ is the respective electron-weighted center of mass and $N = \sum_i n_i$ the total number of electrons. The sum runs over all protein atoms. This value differs marginally from the R_g that would be obtained from the protein electron density,

$$(R_g^{Prot,\rho})^2 = N^{-1} \int \rho(\mathbf{r})(\mathbf{r} - \mathbf{r}_{com})^2 \, d\mathbf{r},$$

because the electrons are spatially distributed around the nuclei. The difference between these two values may be estimated with a simple model: (a) Let a set of electrons be positioned at $\mathbf{R}_0 = (R_0, 0, 0)$, implying that the radius of gyration $R_{g,m}$ with respect to the origin is R_0. (b) Alternatively, let the electrons be distributed as a three-dimensional Gaussian of width σ centered at $\mathbf{R}_0$, where $\sigma \ll R_0$. Then, the radius of gyration is given via

$$(R'_{g,m})^2 = \frac{1}{(2\pi)^{(3/2)}\sigma^3} \int \mathbf{r}^2 \, e^{-(\mathbf{r}-\mathbf{R}_0)^2/2\sigma^2} d^3\mathbf{r} = R_0^2 + 3\sigma^2$$

and the difference in radius of gyration between cases (a) and (b) is $R'_{g,m} - R_{g,m} \approx 3\sigma^2/(2R_0)$. Thus, upon smearing out electrons around the position $\mathbf{R}_0$, the R_g value is slightly increased.

As a numerical example, let $R_0 = 11$ Å, corresponding approximately to the R_g value of the GB3 domain, and $\sigma = 0.4$ Å, modeling an electron density distribution that, very approximately, resembles the electron density distribution of carbon. Then, $R'_{g,m} - R_{g,m} = 0.024$ Å. (A more precise calculation using electron densities from Slater-type orbitals yields

similar values.) To test these estimates, we computed $R_\mathrm{g}^{\mathrm{Prot},\rho}$ from a simulation of GB3, obtained with a Guinier analysis of a SAXS curve computed after setting the form factors of all solvent atoms to zero. We obtained $R_\mathrm{g}^{\mathrm{Prot},\rho} = 11.032\,\text{Å}$, whereas the value from the atomic coordinates was $R_\mathrm{g}^{\mathrm{Prot}} = 11.002\,\text{Å}$. The difference is in reasonable agreement with the simple model, suggesting that the model provides a reasonable order-of-magnitude estimate for the increase of $R_\mathrm{g}^{\mathrm{Prot},\rho}$ relative to $R_\mathrm{g}^{\mathrm{Prot}}$.

Together, this analysis suggests that $R_\mathrm{g}^{\mathrm{Prot}}$ provides a good approximation to $R_\mathrm{g}^{\mathrm{Prot},\rho}$. Thus, in this study, for the sake of simplicity, we used $R_\mathrm{g}^{\mathrm{Prot}}$ to quantify the R_g value of the protein.

Supporting Information Figures

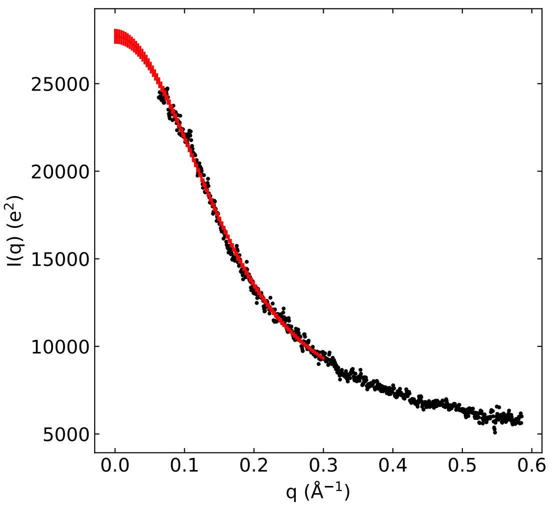

Figure S1: Experimental SAXS data by Zagrovic *et al.*[2] (black dots) and SAXS curve of XAO ensemble obtained by maximum-entropy ensemble refinement (red curve).[1] From the refined XAO ensemble, 20 frames were selected as representative conformations of the heterogeneous XAO ensemble and used subsequently for computing SAXS curves of XAO mutant. The experimental SAXS curve represents the extrapolation to infinite dilution from a set of experiments in 100 mM acetate buffer at 15, 10, 5, 2.5, and 1.25 mg/ml. SAXS data kindly provided by Jan Lipfert.[2]

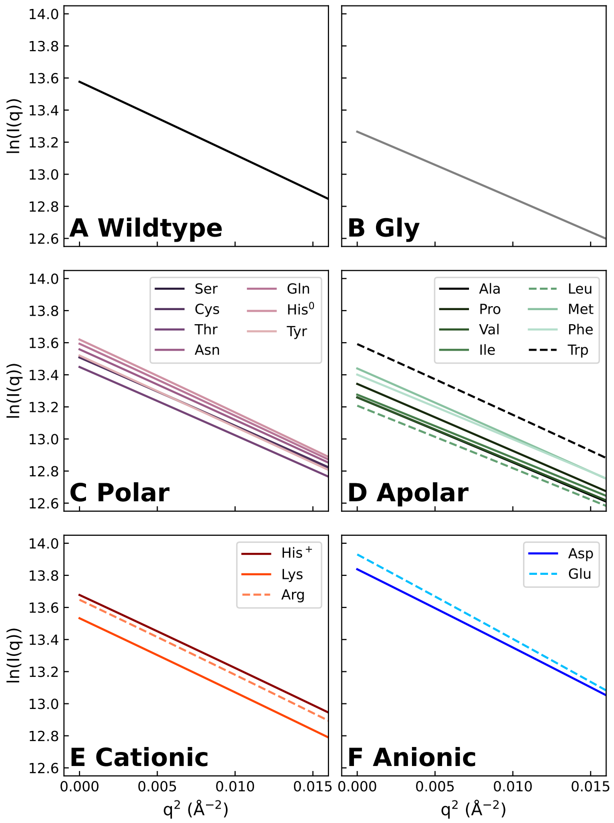

Figure S2: Guinier plots of SAXS curves of the GB3 domain from explicit-solvent SAXS calculations with the TIP4P/2005 water model in combination with the ff03w protein force field. Same SAXS curves as shown in Fig. 2A–F, however plotted as $\ln(I(q))$ vs. q^2.

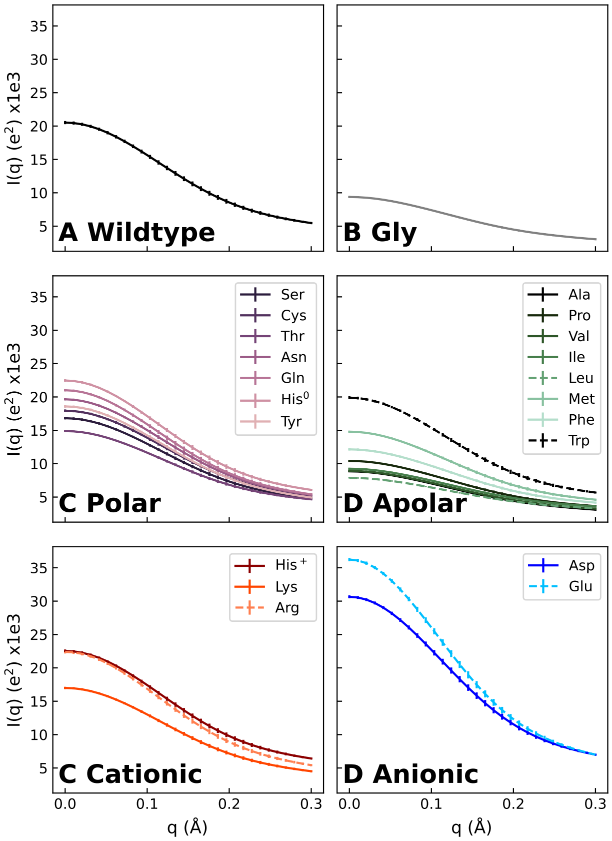

Figure S3: SAXS curves of the heterogeneous ensemble of the XAO peptide from explicit-solvent SAXS calculations with the TIP4P/2005 water model in combination with the ff03w protein force field. Backbone positions were restrained in simulations for all XAO mutants to the backbone positions of the XAO wild type ensemble refined against experimental SAXS data (see Fig. S1), suggesting that variations among the computed SAXS curves are purely caused by presence of four different amino acids (at fixed backbone positions) and by variations of the hydration shell. SAXS curves are shown (A) for the XAO wild type and (B–F) for for 21 mutants with four mutated surface-exposed amino acids each (for color code and line style, see legends). For clarity, SAXS curves are grouped by the amino acid property (glycine, polar, apolar, cationic, anionic) in panels B–F.

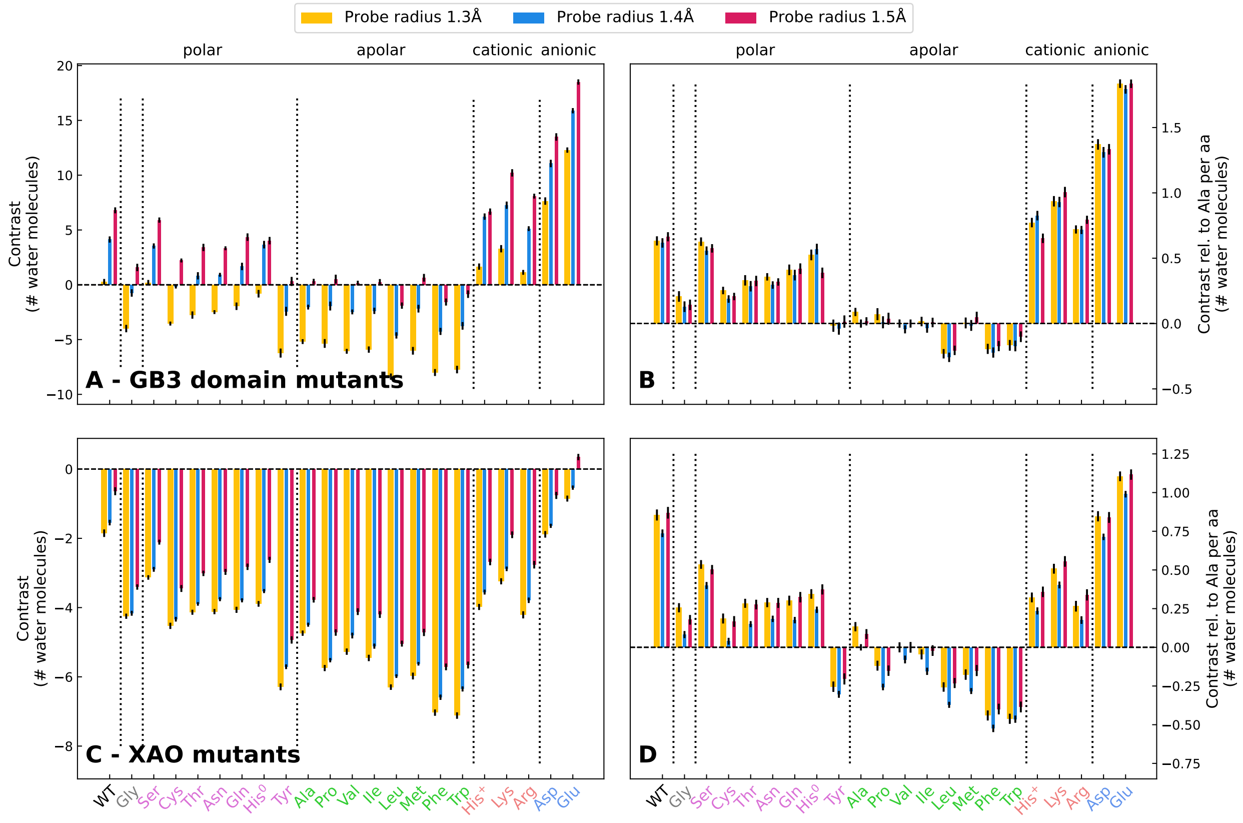

Figure S4: On the effect of using different probe radii for computing the protein volume on computed hydration shell contrasts. (A) Contrast of the hydration shell in number of water molecules of GB3 wild type and 21 GB3 mutants, see labels at abscissa colored by the property of the amino acid: Gly (grey), polar (pink), apolar (green), cationic (orange), anionic (blue) residues. Contrast values were obtained using three different probe radii for computing the protein volume with the 3V volume calculator:[8] 1.3 Å (yellow), 1.4 Å (blue), or 1.5 Å (red). A grid spacing of 0.16Å was used. (B) Contrast per amino acid for GB3 domain relative to alanine. (C/D) Same analysis as in panels (A/B) for the XAO peptide. From a given MD simulation, the total contrast is unambiguously obtained via the forward scattering $I(0)$. However, using larger probe radii lead to larger computed protein volumes, thereby to smaller density contrasts assigned to the bare protein, and, consequently, larger contrasts assigned to the hydration shell as shown in panels (A/C). In contrasts relative to alanine hardly depend on the choice of the probe radius, as shown in panels (B/D).

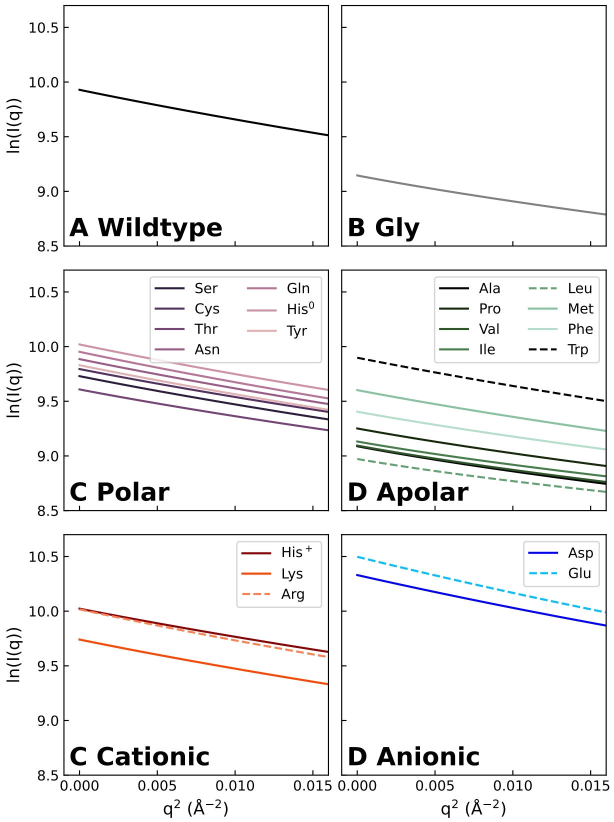

Figure S5: Guinier plots of SAXS curves of the heterogeneous ensemble of the XAO peptide from explicit-solvent SAXS calculations with the TIP4P/2005 water model in combination with the ff03w protein force field. Same SAXS curves as shown in Fig. S3A–F, however plotted as $\ln(I(q))$ vs. q^2.

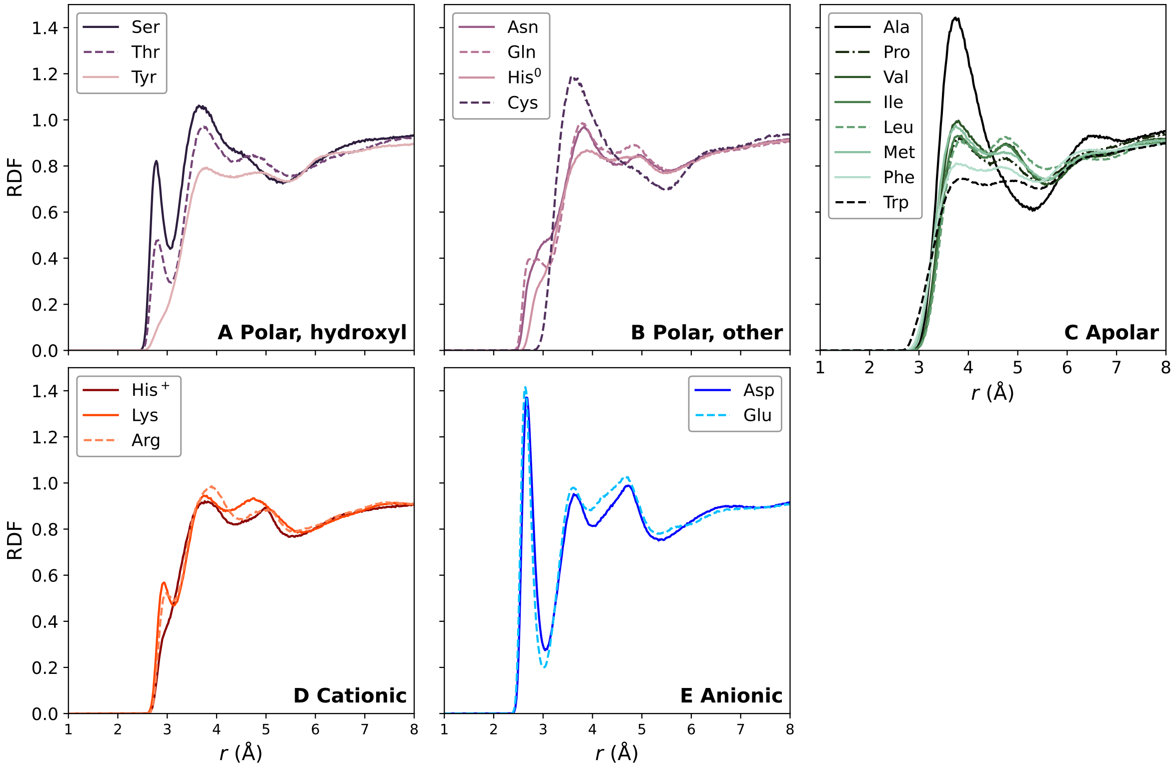

Figure S6: Radial distributions functions (RDFs) of water oxygen atoms with respect to side chain heavy atoms of amino acids computed from simulations of mutated XAO variants. For color code, see legends. RDFs were obtained from simulations with TIP4P/2005 and averaged over 20 XAO conformations. Results from different amino acids are grouped by (A) polar residues containing a hydroxyl group, (B) other polar residues, (C) apolar, (D) cationic, and (E) anionic residues.

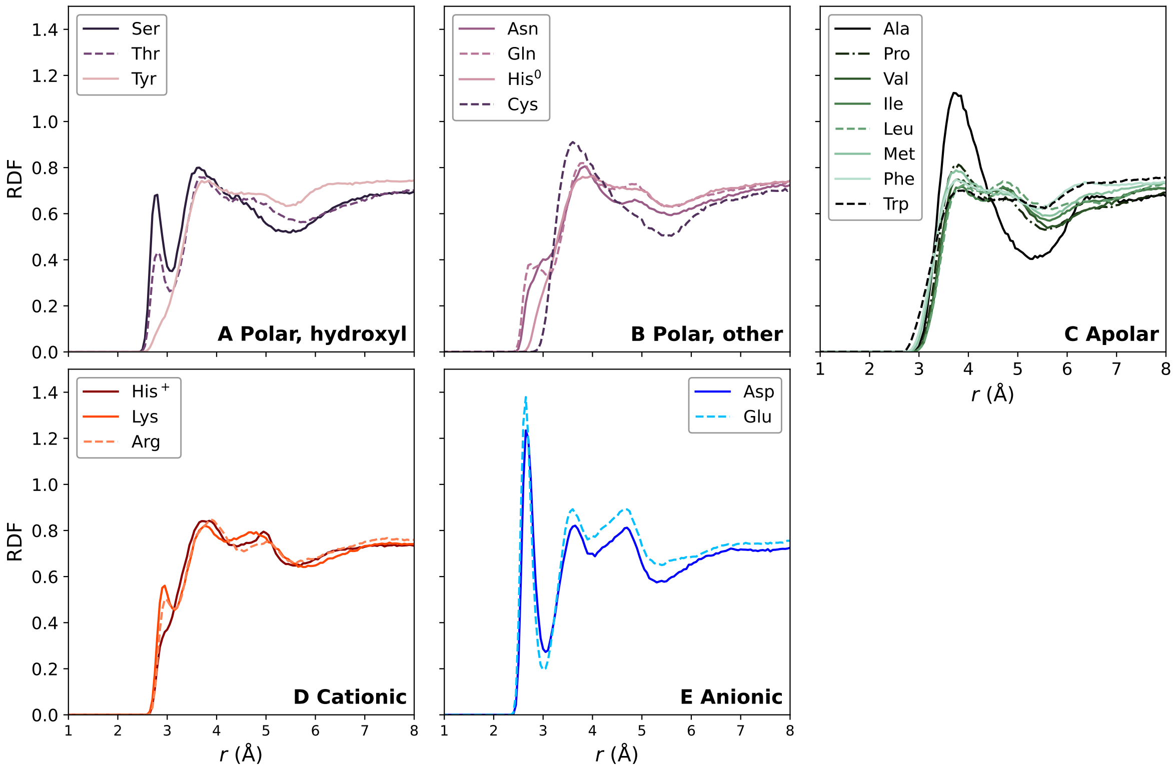

Figure S7: RDFs of water oxygen atoms with respect to side chain heavy atoms of the ten mutated residues of mutated GB3 variants. Presentation analogous to Fig. S6.

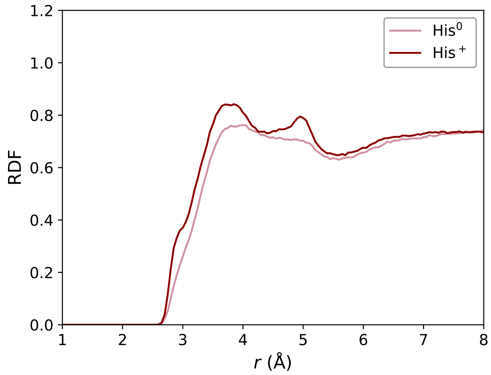

Figure S8: RDFs of water oxygen with respect to side chain heavy atoms of neutral δ-protonated (His^0) and cationic double-protonated histidine (His$^+$). RDFs were computed from simulations of mutated GB3 variants. Water is more tightly packed around His$^+$ compared to His^0.

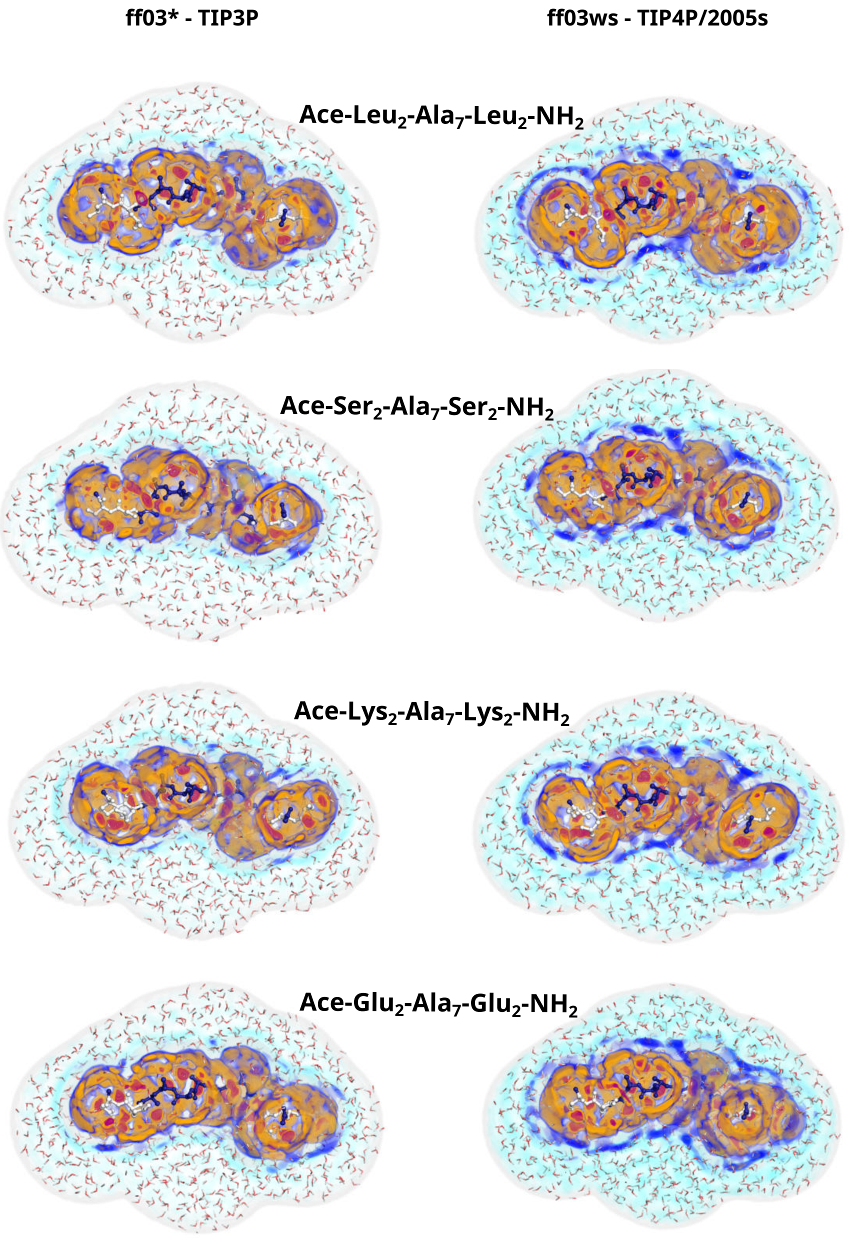

Figure S9: Three-dimensional densities of the hydration shell around the XAO mutants with four leucine, serine, lysine, or glutamate residues at the termini (see labels). The densities were calculated from simulations using ff03* in conjunction with TIP3P (left column) or using ff03ws in conjunction with TIP4P/2005s (right column). Color code is taken from Fig. 1B/D. The solvent densities depend on amino acid type and on the force field.

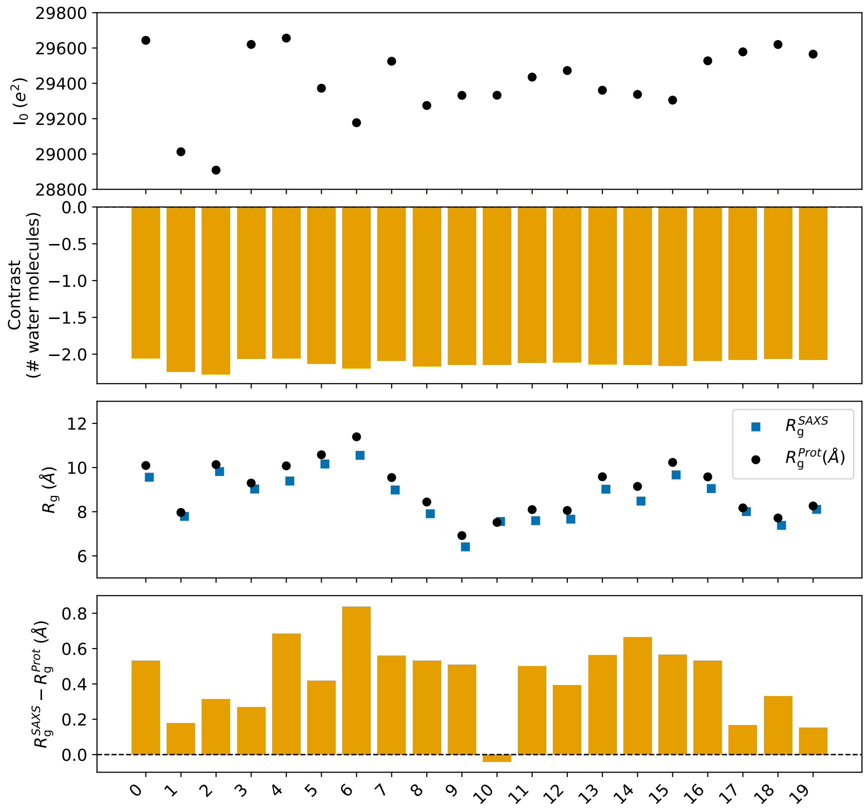

Figure S10: Forward scattering I_0, hydration shell contrast in number of water molecules, R_g, and ΔR_g values for 20 conformations of the aspartate mutant of XAO, obtained with TIP4P/2005 and ff03w. The different conformations impose similar hydration shell contrasts, yet lead to greatly different ΔR_g values.

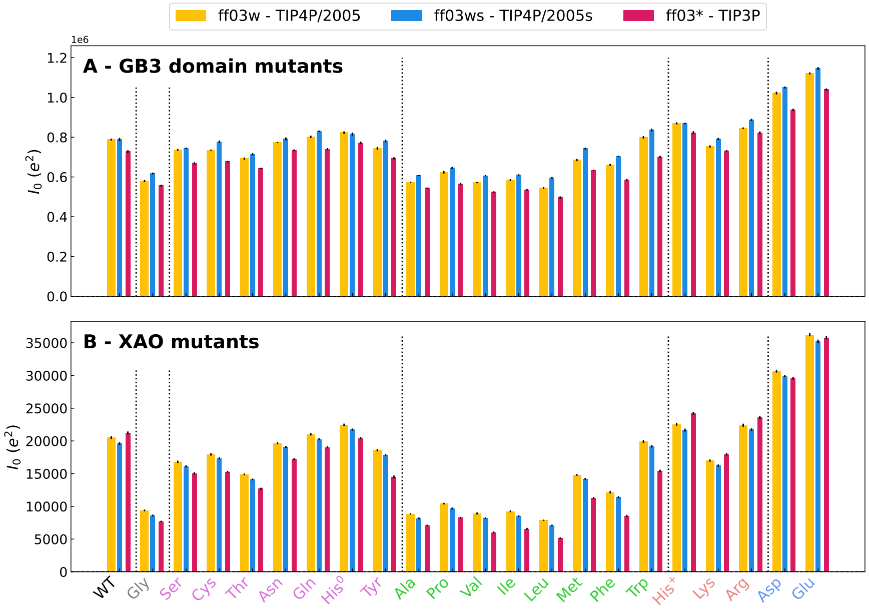

Figure S11: Forward scattering intensity I_0 from SAXS curves for wild type and 21 mutants (see labels on abscissa) of (A) GB3 domain and (B) XAO peptide from simulations with three different combinations of protein force field and water model (for color code, see legend).

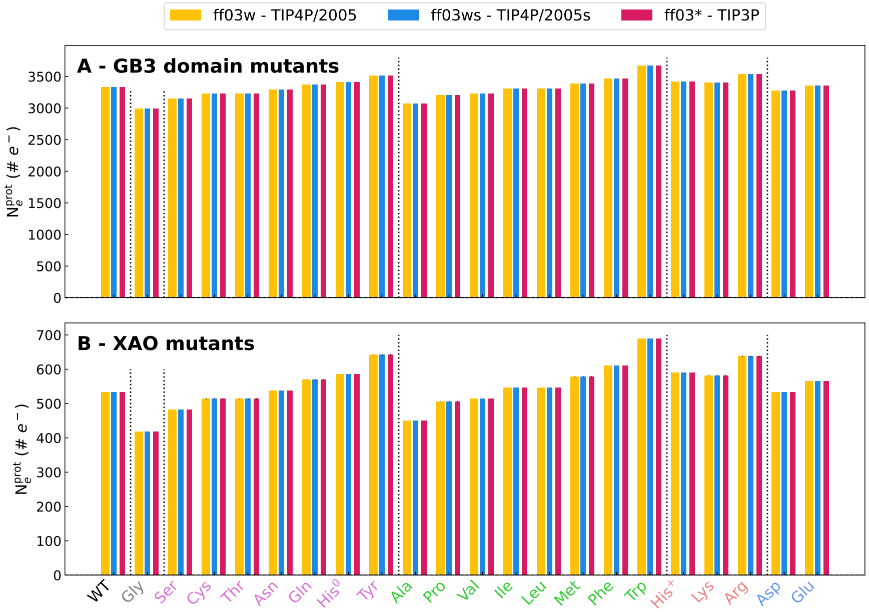

Figure S12: Number electrons (# e^-) of the solute for WT and 21 mutants of (A) GB3 domaind and (B) XAO peptide from simulations with three different combinations of protein force field and water model (for color code, see legend).

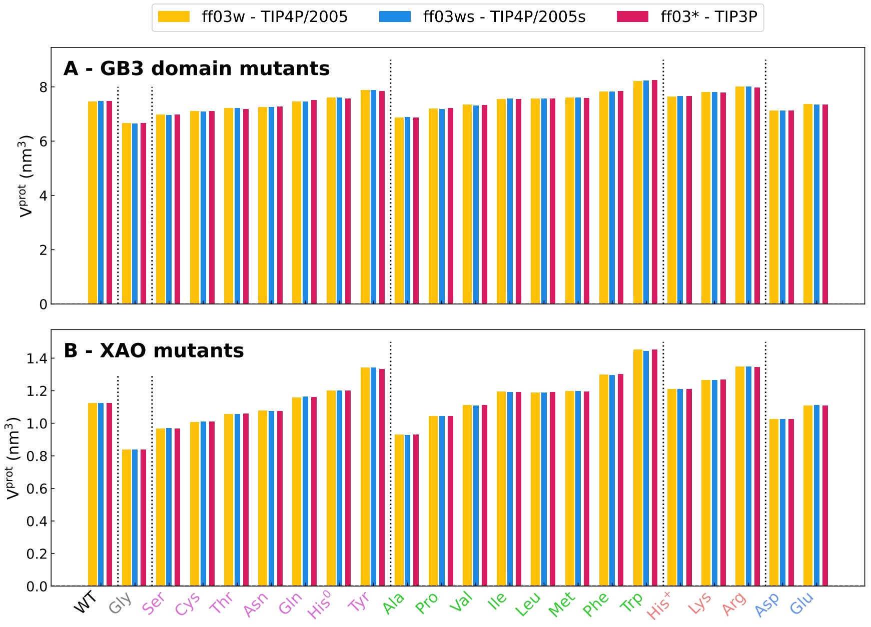

Figure S13: Volumes of WT and 21 mutants (see labels on abscissa) of (A) GB3 domain and (B) XAO peptide from simulations with three different combinations of protein force field and water model (for color code, see legend).

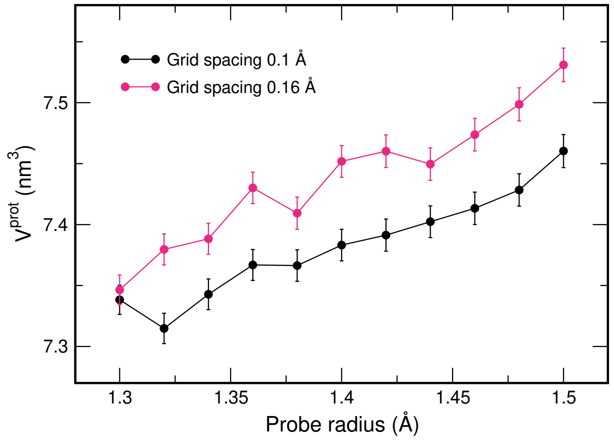

Figure S14: On the effect of using different probe radii or grid spacing for computing the protein volume. GB3 volumes were computed with the 3V volume calculator[8] using different probe radii (abscissa) or using a grid spacing of either 0.1 Å (black) or 0.16 Å (magenta). Error bars denote 1 SE obtained from the average of 20 MD simulation frames. The choices of both probe radius and grid spacing influence the computed protein volumes and, thereby, the absolute contrasts assigned to the hydration shell. In contrast, the contrasts relative to alanine depend only marginally on the choice of the volume definition (see Fig. S4).

References

(1) Hermann, M. R.; Hub, J. S. SAXS-Restrained Ensemble Simulations of Intrinsically Disordered Proteins with Commitment to the Principle of Maximum Entropy. *J. Chem. Theory Comput.* **2019**, *15*, 5103–5115.

(2) Zagrovic, B.; Lipfert, J.; Sorin, E. J.; Millett, I. S.; Van Gunsteren, W. F.; Doniach, S.; Pande, V. S. Unusual Compactness of a Polyproline Type II Structure. *Proc. Natl. Acad. Sci. U.S.A.* **2005**, *102*, 11698–11703.

(3) Makowska, J.; Rodziewicz-Motowidło, S.; Bagińska, K.; Vila, J. A.; Liwo, A.; Chmurzyński, L.; Scheraga, H. A. Polyproline II Conformation Is One of Many Local Conformational States and Is Not an Overall Conformation of Unfolded Peptides and Proteins. *Proc. Natl. Acad. Sci. U.S.A.* **2006**, *103*, 1744–1749.

(4) Knight, C. J.; Hub, J. S. WAXSiS: a web server for the calculation of SAXS/WAXS curves based on explicit-solvent molecular dynamics. *Nucleic Acids Res.* **2015**, *43*, W225–W230.

(5) Chen, P.-c.; Hub, J. S. Validating Solution Ensembles from Molecular Dynamics Simulation by Wide-Angle X-ray Scattering Data. *Biophys. J.* **2014**, *107*, 435–447.

(6) Chen, P.-c.; Hub, J. S. Interpretation of Solution X-ray Scattering by Explicit-Solvent Molecular Dynamics. *Biophys. J.* **2015**, *108*, 2573–2584.

(7) van Gunsteren, W. F.; Berendsen, H. J. C. A Leap-Frog Algorithm for Stochastic Dynamics. *Mol. Sim.* **1988**, *1*, 173–185.

(8) Voss, N. R.; Gerstein, M. 3V: cavity, channel and cleft volume calculator and extractor. *Nucleic Acids Res.* **2010**, *38*, W555–W562.
